# Perceived Autonomy Support and Motivation in Young People: A Comparative Investigation of Physical Education and Leisure-Time in Four Countries

**DOI:** 10.5964/ejop.v15i3.1735

**Published:** 2019-09-27

**Authors:** Istvan Soos, Ibolya Dizmatsek, Jonathan Ling, Adedokun Ojelabi, Jaromir Simonek, Iulianna Boros-Balint, Peter Szabo, Attila Szabo, Pal Hamar

**Affiliations:** a Department of Pedagogy and Methodology, University of Physical Education, Budapest, Hungary; b University of Physical Education, Budapest, Hungary; c Faculty of Health Sciences and Wellbeing, University of Sunderland, Sunderland, United Kingdom; d Department of Physical Education & Sport, Faculty of Education, Constantine the Philosopher University, Slovakia; e Babes-Bolyai University, Cluj-Napoca, Romania; f Eotvos Lorand University, Budapest, Hungary; Webster University Geneva, Geneva, Switzerland

**Keywords:** perceived autonomy support, autonomous motivation, physical education, leisure-time physical activity, young people

## Abstract

Physical education focuses on the development of sports skills as well as fitness for health. In Central European countries there has been a shift in these focuses since the fall of Communism to follow internationally-recognised health-related physical activity recommendations, similar to Western European countries. In this study we investigated the extent to which motivation from school physical education transfers to leisure-time physical activity providing autonomy support by three social agents: school (physical education teachers), family and peers. Our study utilised the Aetological Approach (AA), Ecological Model (EM) and the Trans-Contextual Model (TCM) that consists of the Theory of Planned Behaviour (TPB) and the Self-Determination Theory (SDT) to explore how autonomous motivation is transferred between contexts (physical education, leisure-time and current behaviour). Nine-hundred and seventy-four students aged 11–18 (55% girls) participated in our study from four countries: Hungary, United Kingdom, Romania and Slovakia. A prospective research design was employed, and questionnaires were administered at three time points. Using 7-point Likert scales, attitude, usefulness, and affectiveness were measured. Furthermore, subjective norms and perceived behavioural control (PBC) were tested within TPB. Autonomous and controlling motivation were measured within SDT by administering the Behavioural Regulation in Exercise questionnaires (BREQ and BREQ-2). Finally, past physical activity, intention and current physical activity behaviours were tested. Results indicated that perceived autonomy support from family and friends predicted autonomous motivation towards leisure-time physical activity in all four countries. However, teachers’ behaviour in some Eastern European countries did not predict this transfer. In general, in line with previous literature, boys reported more physical activity than girls. A strong influencing factor in the path model was that past behaviour predicted current behaviour, and according to that factor, boys reported being more active than girls.Boys also perceived more support from PE teachers than girls which was likely to have influenced their autonomous motivation in PE, which in turn transferred to leisure time. We discuss these results in the context of theories exploring the role of motivation and social environment on children’s choices related to physical activity. In conclusion, we suggest providing more autonomy support, especially by schools, for the enhancement of autonomous motivation of young people to promote their leisure time physical activity.

In Eastern European Countries, prior to the fall of Communism, the main aim of school physical education was to improve students’ physical and mental skills, and to prepare them for compulsory national military service. However, since the fall of the Iron Curtain, many changes have occurred in school education, including physical education. While the main emphasis of school physical education is still partly skills development, attention has shifted towards health-related exercise/physical activity. In particular, there may be cross-cultural variations in how behaviour from school physical education is transferred to leisure-time physical activity (Weinberg et al., 2000), such as between predominantly collectivist countries and individualist countries (Hagger et al., 2009), however no previous research has investigated this issue.

In summary, individualist and collectivist cultures can be characterized as follows (see also Fattah, Darwish, & Huber, 2003). Conformity may occur more frequently in collectivist cultures, when the norms are clear and sanctions are likely to be imposed for deviant behaviour. Individualistic cultures emphasize promoting the individual’s self-interest (underlining individual rights, not responsibilities), personal autonomy, privacy, self-realization, independence, an understanding of personal identity as the sum of attributes of the individual, and less concern about the needs and interests of others. The UK, USA and Australia are examples of typical individualistic societies. Collectivism is exemplified by Eastern European countries such as Hungary, Romania and Slovakia.

Positive experiences in school physical education may be transferred to young people’s leisure time (also referred to as free time or free living) which may lead to a physically active lifestyle in later life (Shephard & Trudeau, 2000; Taylor, Blair, Cummings, Wun, & Malina, 1999). Therefore, school physical education has an important role not only in young people’s physical and mental health, but can ultimately affect public health (Sallis & Mckenzie, 1991).

In the past two decades, several researchers have investigated the role of different agents in motivating young people to adopt active lifestyles. These agents may include teachers in delivering an efficient physical education system, and family and friends as role models in young people’s free time. According to Pate et al. (1995) and Taylor et al. (1999), physical education teachers can effectively orient young people toward leisure-time physical activity outside school. Thus, a positive motivational climate adopted by physical education teachers can foster wider positive health-related behaviours whereby in a stimulating environment, students are more likely to adopt autonomous motivation in order to pursue health-related physical activities out of choice in their free time and in the absence of external persuasion (Ames & Archer, 1988; Pakarinen, Parisod, & Smed, 2017).

Several theories, models and approaches have attempted to explain the determinants of young people’s physical activity (or inactivity) as well as the pre-cursors (mediators) of these, such as how motivation and other factors can influence intention that finally becomes the actual behaviour. In this study, we explore the relationship between these determinants for the most commonly used approaches.

- The Aetiological Approach (Hagger & Chatzisarantis, 2016) describes both the correlational relationships (in which factors are associated with activities), as well as the determinants of intentions, decision making and physical activity behaviour. We investigated these relationships by examining the perceived autonomy support path to leisure-time physical activity intention and behaviour through the antecedents of autonomous motivation both in physical education and leisure-time contexts.
- The EM (Baumann et al., 2012) describes not only psychological and biological, but interpersonal determinants, such as social support (from family, friends and work or school), and cultural norms and practices. The environmental determinants comprise the social, built and natural environments. These factors are linked to a range of policies, including parks and recreation, health, education, organised sports and national physical activity advocacy. Finally, global determinants involve factors from economic development to social and cultural norms.
- The TPB attempts to explain the volitional antecedents of intentional behaviour (Ajzen, 1985) with intention argued to be the strongest predictor of behaviour (Armitage & Conner, 2001), and to summarize people’s general affective and cognitive orientation toward a specific behaviour (Hagger, Chatzisarantis, Culverhouse, & Biddle, 2003). TPB comprises three constructs, attitude, subjective norm and PBC. Attitude reflects an individual’s personal orientation toward engaging in the behaviour. Subjective norms reflect social pressure from significant others regarding to the behaviour. PBC reflects the impact of perceived abilities and barriers with respect to engaging in the behaviour (Hagger, Chatzisarantis, Barkoukis, Wang, & Baranowski, 2005).
  While much research has supported the basic principles of TBP (Hamilton & White, 2008), Ryan and Deci (2000) found that if subjective norm is perceived as social pressure, then it impedes rather than enhances motivation. They suggest implementing non-pressurising forms of social influence, such as providing young people with choice (significant others encourage participation in decision-making), rationale (significant others explain why participating in an activity is important), and acknowledgement of intrapersonal conflict (significant others acknowledge personal feelings and perspectives). Ultimately, these three factors should facilitate perceived autonomy support and hence autonomy support facilitates internalization of the behaviour, so that beliefs, attitudes and emotions are consistent with the behaviour (Chatzisarantis, Hagger, & Smith, 2007).
- The SDT consists of three basic needs to satisfy, the need for autonomy (the belief that one is the origin and regulator of his or her own action), competence (the belief that one can efficaciously interact with the environment), and relatedness (the seeking and development of secure and connected relationships with others in one’s social context) (Deci & Ryan, 1985). The perceived locus of causality (PLOC) is viewed as a continuum of motivation, and a focal point to SDT that draws a distinction between autonomous and non-autonomous (controlled) motivation (Ryan & Connell, 1989). The origin or causes of the behaviour can be either internal, intrinsic motivation, or from external sources, demands or expectations—extrinsic motivation. According to Vallerand’s (1997) Hierarchical Model of Motivation (HMM) the three qualities of intrinsic motivation are intrinsic motivation to know (engagement in an activity to exploring and attempting to understand something new), intrinsic motivation toward accomplishments (engagement in an activity for the satisfaction and pleasure experienced when attempting task mastery or in creating something new), and intrinsic motivation to experience stimulation (engagement in an activity for feeding of sensory pleasure, fun, excitement and aesthetic enjoyment). Overall, Vallerand’s model incorporates the fundamental tenets of SDT (Deci & Ryan, 1985) and contends that motivation operates at three levels, first the global (or personality), second the contextual (or life domain) and finally, third, situational (or state) levels (Vallerand, 1997, 2001).
- The TCM is an integrated model of motivation incorporating special aspects of the SDT (Deci & Ryan, 1985, 2002), the Hierarchical Model of Motivation (HMM; Vallerand, 1997, 2007) and TPB (Ajzen, 1985, 2002).The TCM is a theoretical model which explains how autonomous motivation transfers across contexts, for example adolescents’ autonomous motivation in leisure time can be determined by autonomous motivation in PE (Barkoukis, Hagger, Lampbropoulos, & Tsorbatzoudis, 2010). Further, Hagger and Chatzisarantis (2007) proposed the premise of the TCM, namely that autonomy support in an educational context (e.g., PE) is related to autonomous motivation in an educational context which is in turn transferred to autonomous motivation in a non-educational context (e.g., leisure time). This effect might be especially enhanced if the leisure time behaviour is also reinforced, while significant others support autonomy.

We aimed to investigate the link between perceived autonomy support from three social agents (school, family and friends) and autonomous motivation in an educational context (physical education) as well as leisure-time physical activity. We also examined how perceived autonomy support can be translated into leisure-time physical activity intention and behaviour as based on SDT, TPB and the TCM. Like previous studies (Hagger et al., 2009), we compared results from collectivist countries (Hungary, Slovakia and Romania) with a country (United Kingdom) where historically individualist cultural norms are dominant (Markus & Kitayama, 1991). Previous research has reported consistent differences in the physical activity of boys and girls, with boys tending to be more active. We anticipated that the reason for this could come from motivation to participate, rather than any differences in physical maturation (Sherar, Esliger, Baxter-Jones, & Tremblay, 2007) and so would predict higher scores for boys on a range of measures related to motivation.

Hypotheses:

1. National differences were predicted in SDT (perceived autonomy support by three social agents) as well as in TPB (attitude, subjective norm and PBC) elements within the TCM as those from individualistic countries are hypothetized as enjoying more autonomy than students of collectivist countries.
2. Gender differences were predicted in SDT (perceived autonomy support by three social agents) as well as in TPB (attitude, subjective norm and PBC) elements within the TCM.
3. Positive inter-correlation links were hypothesized between (a) autonomous motivation in PE and leisure-time, as well as between (b) SDT (perceived autonomy support from three social agents: school, family and friends) and TPB (attitude, subjective norm and PBC) elements within the TCM. These factors ultimately influence (c) intention and determine physical activity behaviours.

## Method

### Participants

National research co-ordinators invited schools to participate in the project, and a convenience sample by voluntary participation from various regions were included in the study. The sample was stratified on the basis of age, type of school and geographical region. Sampling was led by local co-ordinators, who contacted schools and recruited participants on a voluntary basis. Inclusion criteria comprised healthy school students, at least 11 years of age, but no more than 18 years of age.

The mean age of participants was 15 years (*SD* = 1.79; range 11.0–18.0 years. Frequencies and percentages of participants are shown in Table 1.

**Table 1:** Number of Participants by Country and Gender

Country	Boys		Girls		Total	
	*N*	%	*N*	%	*N*	%
UK	87	56	68	44	155	16
Hungary	113	29	276	71	389	40
Romania	99	54	84	46	183	19
Slovakia	110	45	137	55	247	25
Total	409	42	565	58	974	100

### Instruments

Instruments were employed according to the TPB (Ajzen, 1985) and SDT (Deci & Ryan, 1985). We followed Hagger et al.’s (2009) study protocol, which also reported the validity of the instruments used in this study. Reliability of our questionnaires is reported in the Results.

The TPB measures (Ajzen & Madden, 1986; Courneya & McAuley, 1994) assessed attitudes based on moral evaluations (bad-good), instrumental evaluation (useful-useless, harmful-beneficial) and affective evaluation (enjoyable-unenjoyable, interesting-boring). Subjective norms were measured by three items on 7-point Likert-scales, ranging from 1 (extremely unlikely) to 7 (extremely likely). An example was “Most people close to me expect me to do active sports and/or vigorous exercise for at least 20 minutes, 3 days per week during my free time over the next 4 weeks.” PBC was assessed through three items on 7-point Likert scales, ranging from 1 (strongly disagree) to 7 (strongly agree). An example was “If I wanted to I could do active sports and/or vigorous exercise for at least 20 minutes, 3 days per week during my free time over the next 4 weeks.” The focal point of SDT is a continuum of autonomous motivation to controlling motivation, known as the PLOC (Ryan & Connell, 1989) and measured by the Behavioural Regulation in Exercise Questionnaire (Mullan, Markland, & Ingledew, 1997) both in physical education and leisure-time contexts. The items measure intrinsic regulation (e.g., “I exercise because I enjoy my exercise sessions”), identified regulation (e.g., “I exercise because I value the benefits of exercise”), introjected regulation (e.g., “I exercise because I will feel bad about myself when I don’t exercise”), and exercise regulation (e.g., “I exercise because people important to me, parents, family, etc., say I should”) on 7-point Likert scales ranging from 1 (not true at all for me) to 7 (very true for me).

Past physical activity behaviour was also measured in accordance with previous studies (Hagger et al., 2003) by using one item: “I engaged in active sports and/or vigorous activity for 20 minutes at a time the in the past 4 weeks with following regularity.” Participants were asked to indicate the frequency of their past active sport and/or physical activity participation during their free time on a six-point Likert-scale, anchored by not at all (1) and most of the days during the week (6).

Self-reported physical activity was assessed in the third wave of data collection by an adaptation of the Leisure-Time Exercise Questionnaire (Godin & Shephard, 1985). The 12-item Perceived Autonomy Support Scale for Exercise Settings (PASSES; Hagger et al., 2007) was also administered in order to measure participants’ perceived autonomy support in physical education, family and friends settings. An example item from the scale is “I feel that my physical education teacher provides me with choices, options, and opportunities to do active sports and/or vigorous exercise.” Regarding family context, the words “physical education teacher” were replaced with “family” in all items, and in the third scale regarding friends context, “family” was replaced with “friends” in all items.

Intentions were measured by three items (e.g., I intend to do active sports and/or vigorous physical activity during my leisure time in the next 5 weeks) rated on 7-point Likert-scales ranging from 1 (strongly agree) to 7 (strongly disagree).

### Translation Procedure

A translation procedure was employed according to Brislin (1986). Bilingual translators drafted the questionnaires in the language of the county where data collection was taken place, which was back-translated into English by two independent translators. The two versions were compared, and any inconsistencies, errors, biases and incongruences were corrected, with further translation and back translation was conducted until the two versions were identical (see Bracken & Barona, 1991). The final version was administered to participants in the study.

### Procedure

Ethical approval was granted by the Research Ethics Committee at the Principal Investigator’s university. Consent forms were signed both by parents and participants. All ethics met the Helsinki Declaration (Harris & Atkinson, 2009).

Data collection followed a three-wave prospective design. For the first phase, participants completed a short questionnaire of their physical activity in the past 6 months, the Perceived Autonomy Support Scale (Hagger et al., 2007) for physical education, the PLOC in a physical education context, and their intention for leisure-time physical activity in the future. One week later, participants completed a short attitude scale in a quiet classroom environment, a subjective norm scale and social norm scale towards physical activity behaviour and the PLOC in a leisure-time physical activity context. In addition, they completed the Perceived Autonomy Support Scale (Hagger et al., 2007) both for family and friends. Finally, 4 weeks later participants completed a short scale on their current participation in physical activity, under the same quiet classroom environment as they did 4 weeks earlier (Godin & Shephard, 1985).

### Data Analysis

IBM SPSS v.24 and AMOS v.24 (Arbuckle, 2016) software package were employed for data analysis. After preliminary analyses (reliability analyses, calculation and the Relative Autonomy Index (RAI), descriptive statistics, Pearson correlations, independent samples *t*-tests and univariate analysis of variance [ANOVA]), path analyses were performed for each country separately as well as for the full data set. In this study we report the combined model. Significance was set at *p* < .05.

## Results

### Preliminary Analyses

#### Reliability Analysis

Reliability analysis indicated that the reliabilities for all scales were satisfactory: attitude α = .895, perceived behavioural control α = .872, subjective norm α = .755, PASSES PE teacher α = .952, PASSES family/parents α = .958, PASSES peers α = .943, External regulation PE α = .697, introjected regulation PE α = .823, identified regulation PE α = .800, intrinsic regulation PE α = .801, external regulation leisure time α = .819, identified regulation leisure time α = .816, intrinsic regulation leisure time α = .886, and intention α = .898.

#### Relative Autonomy Index (RAI)

According to the PLOC constructs (PLOC; Guay, Magean, & Vallerand, 2003), weights were assigned to each individual’s intrinsic regulation (+2), identified regulation (+1), introjected regulation (−1) and extrinsic regulation (−2). RAI was the composite of the weighted scores and reflected participants’ autonomous motivation both in physical education (PE) and leisure-time physical activity contexts. We employed this protocol as The RAI provides a direct measure of motivational autonomy (Vaz, Pratley, & Alkire, 2016).

Preliminary analysis indicated that there were no significant age differences found so we did not explore this variable further.

### National Differences

*Hypothesis 1: National differences were predicted in SDT as well as in TPB elements within the TCM*.

Univariate ANOVA showed that there were significant differences (*p* < .001) between all the four countries (see Table 2) in perceived autonomy support from teachers, *F*(3, 970) = 307.455, perceived autonomy support from family, *F*(3, 970) = 40.758, perceived autonomy support from friends, *F*(3, 970) = 14.378, attitude to exercise, *F*(3, 970) = 8.051, subjective norm, *F*(3, 970) = 19.419, PBC, *F*(3, 970) = 22.176, past physical activity behaviour, *F*(3, 970) = 14.974, intention for doing physical activity, *F*(3, 970) = 12.883, current physical activity behaviour, *F*(3, 970) = 7.449, autonomous motivation for physical education (AMPE), *F*(3, 970) = 31.786, and autonomous motivation for leisure-time physical activity (AMLT), *F*(3, 970) = 5.508.

**Table 2:** Descriptive Statistics for Gender, Country and Age Groups by TPB Construct

Group	PAS teachers		PAS peers		PAS family		Attitude		Subjective norm		PBC		Past behaviour		Intention		PA behavior		AMPE		AMLT	
	*M*	*SD*	*M*	*SD*	*M*	*SD*	*M*	*SD*	*M*	*SD*	*M*	*SD*	*M*	*SD*	*M*	*SD*	*M*	*SD*	*M*	*SD*	*M*	*SD*
Boys	4.39	1.50	4.70	1.30	4.87	1.25	5.70	1.21	5.02	1.21	5.37	1.33	3.87	1.53	5.08	1.64	3.76	0.75	-2.05	2.14	5.93	5.08
Girls	4.14	1.29	4.98	1.18	5.20	1.21	5.79	0.97	5.24	1.08	5.61	1.14	3.41	1.38	5.10	1.55	3.66	0.58	-1.03	2.17	6.90	4.89
Hungary	4.61	1.21	5.10	1.14	5.48	1.14	5.81	0.88	5.36	1.06	5.69	0.97	3.58	1.51	5.41	1.50	3.72	0.52	-1.42	2.26	7.06	5.07
UK	5.22	1.06	4.86	1.29	5.03	1.39	5.93	1.13	5.36	1.21	5.49	1.34	4.24	1.38	5.09	1.44	3.86	0.89	-2.89	2.33	5.54	4.94
Romania	2.89	0.38	4.91	1.16	5.07	1.15	5.84	1.25	5.09	1.12	5.81	1.31	3.58	1.43	5.03	1.48	3.73	0.65	-1.10	1.86	6.98	5.02
Slovakia	4.50	0.89	4.46	1.14	4.42	1.08	5.47	1.16	4.71	1.12	4.99	1.30	3.26	1.33	4.62	1.76	3.56	0.64	-0.88	1.97	5.86	4.73
Younger^a^	4.42	1.36	4.91	1.27	5.22	1.22	5.88	1.01	5.32	1.17	5.62	1.19	3.87	1.44	5.23	1.51	3.78	0.69	-1.77	2.25	5.96	5.11
Older^b^	4.09	1.40	4.82	1.22	4.92	1.24	5.63	1.13	4.99	1.1	5.41	1.25	3.36	1.44	4.96	1.64	3.63	0.61	-1.18	2.15	6.98	4.83

*Note.* PAS = perceived autonomy support; PBC = perceived behaviour control; AMPE = autonomous motivation in physical education; AMLT = autonomous motivation in leisure time.

^a^Age 11–14. ^b^Age 15–18.

Post hoc tests (with Bonferroni correction) revealed that UK students perceived the most autonomy support from PE teachers, compared with Hungary (*p* < .001), Slovakia (*p* = .001) and Romania (*p* = .001). Hungarian students perceived the most autonomy support from family, compared with UK (*p* = .001), Slovakia (*p* = .001) and Romania (*p* = .001). Also, Hungarian students perceived the most autonomy support from friends, compared to Slovakia (*p* = .001. UK students had the highest level of attitude to exercise, compared with Slovakia (*p* = .001). UK students demonstrated higher subjective norm than Slovakia (*p* = .001) and Romania (*p* = .028). PBC was higher in Romania than UK (*p* = .015) and Slovakia (*p* = .001). The level of past physical activity behaviour was the highest in the UK, compared with Hungary (*p* = .001), Slovakia (*p* = .001) and Romania (*p* = .001). However, the intention to perform physical activity was higher in Hungary than Slovakia (*p* = .001) and Romania (*p* = .007). UK students demonstrated the highest current physical activity behaviour, as opposed to Slovakia (*p* = .001). Slovakia showed higher AMPE than Hungary (*p* = .002) and UK (*p* = .001). Finally, Romania showed higher AMLT than UK (*p* = .049).

Hypothesis 1 is partially accepted.

### Gender Differences

*Hypothesis 2: Gender differences were predicted in SDT as well as in TPB elements within the TCM*.

#### Overall sample (all 4 countries)

Perceived autonomy support from physical education teachers, *F*(1, 972) = 14.249, *p* < .001 (boys), past physical activity behaviour, *F*(1, 972) = 23.820, *p* < .001 (boys), and current physical activity behaviours, *F*(1, 972) = 5.136, *p* = .024; all were higher in boys as opposed to girls.

Perceived autonomy support from family, *F*(1, 972) = 17.167, *p* < .001 (girls); perceived autonomy support from friends, *F*(1, 972) = 12.274, *p* < .001 (girls), subjective norm, *F*(1, 972) = 8.579, *p* = .003 (girls), perceived behaviour control (PBC), *F*(1, 972) = 8.921, *p* = .003 (girls), autonomous motivation in PE (AMPE), *F*(1, 972) = 52.462, *p* < .001, and AMLT, *F*(1, 972) = 9.280, *p* = .002, were higher in girls than boys. There were no other significant difference between girls and boys.

#### Hungary

Perceived autonomy support from physical education teachers, *t*(387) = 6.241, *p* < .001, and past physical activity behaviour, *t*(387) = 2.16, *p* < .001, were higher in boys. PBC, *t*(387) = -2.239, *p* = .026, AMPE, *t*(387) = -4.688, *p* < .001, and AMLT, *t*(387) = -2.241, *p* = .026, were higher in girls. There were no significant differences between girls and boys for the other variables.

#### United Kingdom

Most of the variables were similar, with only perceived autonomy support from PE teachers, *t*(153) = 4.803, *p* < .001, was higher in boys, and AMPE, *t*(153) = -1.998, *p* = .047, was higher in girls.

#### Slovakia

Only past physical activity behaviour, *t*(181) = 4.121, *p* < .001, was higher in boys. Perceived autonomy support from family, *t*(181) = -2.048, *p* = .042, subjective norm, *t*(181) = -2.432, *p* = .016, and AMPE, *t*(181) = -4.911, *p* < .001, were higher in girls.

#### Romania

Girls had higher values than boys in all the following parameters: perceived autonomy support from family, *t*(245) = -3.047, *p* = .003, perceived autonomy support from peers, *t*(245) = -2.806, *p* = .006, subjective norm, *t*(245) = -2.109, *p* = .036, PBC, *t*(245) = -3.568, *p* < .001, AMPE, *t*(245) = -2.154, *p* = .033, and AMLT, *t*(245) = -2.241, *p* = .026.

Hypothesis 2 is therefore accepted.

#### Inter-Correlations Between Model Components

*Hypothesis 3: Positive inter-correlation links between (a) autonomous motivation in PE and leisure-time, (b) SDT and TPB and (c) intention and physical activity*.

Hypothesis 3 which hypothesised that positive inter-correlation links were between (a) autonomous motivation in physical education and leisure-time, (b) SDT (Perceived autonomy support from 3 social agents: school, family and friends) and (c) TPB (attitude, subjective norm and PBC) elements was also accepted. Details of these results are presented in Table 3.

**Table 3:** Descriptive Statistics and Inter-Correlations Among the Extended Trans-Contextual Model Components

Factor	*M*	*SD*	1	2	3	4	5	6	7	8	9	10
1. PAS (PE Teacher)												
Hungary	4.61	1.21										
UK	5.22	1.06										
Romania	2.29	0.38										
Slovakia	4.50	0.89										
2. Autonomous Motivation (PE)												
Hungary	−1.42	2.26	-.50**									
UK	−2.90	2.23	-.29**									
Romania	−1.10	1.86	-.06									
Slovakia	−0.88	1.97	-.26**									
3. PAS (Peer)												
Hungary	5.10	1.26	.23**	-.25**								
UK	4.86	1.29	.29**	-.18*								
Romania	4.91	1.16	-.23**	-.02								
Slovakia	4.46	1.14	.49**	-.17**								
4. PAS (family)												
Hungary	5.48	1.14	.22**	-.27**	.72**							
UK	5.03	1.39	.34**	-.17*	.77**							
Romania	5.07	1.15	-.26**	-.01	.76**							
Slovakia	4.42	1.08	.46**	-.16*	.65**							
5. Autonomous Motivation (LT)												
Hungary	7.05	5.07	.12*	-.41**	.38**	.42**						
UK	5.54	4.94	.25**	-.45**	.36**	.42**						
Romania	6.98	5.02	-.07	-.07	.32**	.31**						
Slovakia	5.86	4.73	.35**	-.47**	.37**	.33**						
6. Attitude												
Hungary	5.81	0.88	.26**	-.28**	.54**	.64**	.42**					
UK	5.93	1.13	.39**	-.36**	.51**	.60**	.48**					
Romania	5.84	1.25	-.32**	-.02	.43**	.47**	.43**					
Slovakia	5.47	1.16	.35**	-.23**	.41**	.44**	.50**					
7. Subjective norm												
Hungary	5.36	1.06	.18**	-.20**	.55**	.69**	.25**	.55**				
UK	5.36	1.21	.35**	-.25**	.64**	.76**	.40**	.58**				
Romania	5.09	1.12	-.20**	.12	.54**	.61**	.10	.41**				
Slovakia	4.71	1.12	.30**	-.15*	.52**	.62**	.27**	.49**				
8. PBC												
Hungary	5.69	0.97	.23**	-.27**	.49**	.57**	.36**	.68**	.57**			
UK	5.49	1.34	.32**	-.32**	.55**	.65**	.53**	.64**	.75**			
Romania	5.81	1.31	-.17*	.10	.34**	.46**	.36**	.58**	.45**			
Slovakia	4.50	1.30	.33**	-.27**	.53**	.44**	.51**	.51**	.52**			
9. Intention												
Hungary	5.41	1.50	.16**	-.27**	.50**	.51**	.41**	.41**	.49**	.34**		
UK	5.09	1.44	.27**	-.21**	.53**	.63**	.35**	.50**	.51**	.52**		
Romania	5.03	1.48	-.31**	.04	.36**	.39**	.35**	.54**	.39**	.47**		
Slovakia	4.62	1.76	.31**	-.34**	.48**	.41**	.53**	.59**	.50**	.64**		
10. Behaviour												
Hungary	3.72	0.52	.09	-.04	.08	.05	.05	.02	.05	.10*	.09	
UK	3.86	0.89	.04	-.01	.30**	.23**	.12	.18*	.21**	.23**	.29**	
Romania	3.73	0.65	-.04	.11	.03	.14*	-.03	.12	.12	.05	.11	
Slovakia	3.56	0.64	.15*	-.06	.16*	.19**	.12	.13*	.01	.25**	.25**	
11. Past Behaviour												
Hungary	3.58	1.51	.21**	-.25**	.40**	.38**	.30**	.40**	.36**	.32**	.60**	.13*
UK	4.24	1.38	.20*	-.14	.31**	.36**	.21**	.33**	.30**	.43**	.60**	.32**
Romania	3.58	1.43	-.17*	-.02	.23**	.21**	.24**	.36**	.29**	.28**	.53**	.28**
Slovakia	3.26	1.33	.19**	-.30**	.24**	.23**	.37**	.37**	.33**	.46**	.63**	.20**

*Note.* For Hungarian sample: *N* = 389; UK sample: *N* = 155; Romanian sample: *N* = 183; Slovakian sample: *N* = 247; PAS = Perceived autonomy support; PE = Physical Education context; LT = Leisure-Time context; PBC = Perceived behavioural control.

*p < .05. **p < .01.

Having compared the gender differences by country, we found that perceived autonomy support in PE was higher in UK and Hungarian boys than girls. In Slovakia and Hungary past physical activity behaviour was higher in boys than girls. In contrast with the other two countries, in Romania and Hungary both AMPE and AMLT were higher in girls than boys. Slovakian and Romanian girls perceived higher autonomy support from family than boys perceived.

### Main Analyses

Data were analysed by path analysis, followed this protocol according to previous studies (Barkoukis et al., 2010; Hagger et al., 2003; Hagger et al., 2009), using a simultaneous process with IBM SPSS AMOS v24 software (Ingram, Cope, Harju, & Wuensch, 2000) and a robust maximum likelihood (ML) estimation method. Composites of the study were averaged and goodness of fit of the proposed model with the data was evaluated using multiple recommended indices of good fit. ML attempts to maximize the likelihood that obtained values of the criterion variable will be correctly predicted. CMIN compares the tested model and the independence model to the saturated model. CMIN/DF, the relative chi-square, is an index of how much the fit of data to model has been reduced by dropping one or more path. GFI, the goodness of fit index, is the proportion of the variance in the sample variance- covariance matrix accounted for by the model. Values exceeding .9 are indicative of a good model. The Normed Fit Index (NFI) is the difference between the two model’s chi-squares divided by the chi-square for the independent model. Values approaching .95 for the NFI and CFI are indicative of an acceptable model. The Comparative Fit Index (CFI) uses a similar approach (with a non-central chi-square) to NFI and is a good index for use even with a smaller sample. The Root Mean Square of Approximation (RMSEA) estimates the lack of fit compared to the saturated model. Values of .05 or less indicates good fit, and .08 or less adequate fit (Hu & Bentler, 1999).

#### Path Analysis

Data were analysed by path analysis (Table 4) using a simultaneous process with IBM SPSS AMOS v24 software and a robust ML estimation method. Averaged composites of the study variables were computed prior to analyses. Goodness of fit of the proposed model with the data was evaluated using multiple recommended indices of good fit: CMIN, NFI, CFI, and RMSEA.

**Table 4:** Standardised Parameter Estimates and Univariate Comparisons From the Path Analyses of the Extended Trans-Contextual Model for Each National Sample

	Sample			
Relationship	Hungary	UK	Romania	Slovakia
PAS (PE teacher) → Autonomous motivation (PE)	-.500**^abc^	-.221*^b^	-.062	-.192
PAS (PE teacher) → Autonomous motivation (LT)	-.183**^b^	-.032	-.006	.056
Autonomous motivation (PE) → Autonomous motivation (LT)	-.425**^bc^	-.373**^b^	.043	-.388
PAS (Peer) → Autonomous motivation (LT)	.095*	.051	.086	.270*^abd^
PAS (Peer) → Attitude	.530**^a^	.801**^c^	.413**^ad^	.448**
PAS (Peer) → Subjective norm	.540**	.911**^bcd^	.565**	.476**
PAS (Peer) → PBC	.452**^abc^	.031	-.196*	.136
PAS (family) → Autonomous motivation (LT)	.309**	.394**	.322**	.127^abd^
PAS (family) → Attitude	.694**^a^	-.232*^b^	.724**	.605**^ab^
PAS (family) → Subjective norm	.568**^abc^	-.164	.143	-.056
PAS (family) → PBC	-.044	.299*^bd^	.122	.157
Autonomous motivation (LT) → Attitude	.386**^c^	.507**	.455**	.532**
Autonomous motivation (LT) → Subjective norm	.067	.048	-.157*^c^	-.009
Autonomous motivation (LT) → PBC	.696**^bc^	.696**^bc^	.218**	.185
Autonomous motivation (LT) → Intention	.283*^bc^	-.006	.110	.119
Autonomous motivation (LT) → Behaviour	.101	.147	.014	.121
Attitude → Intention	.108a	.296*	.206*	.185
Subjective norm → Intention	.444**^bc^	.385**^bc^	.151*	.065
PBC → Intention	-.095	-.112	.153*	.260*^d^
Intention → Behaviour	.052	.199	-.084	.210
PBC → Behaviour	.152*^abc^	.097	.008	.055
Past behaviour → PAS (PE teacher)	.213**^b^	.209*^b^	-.171	-.271*^b^
Past behaviour → Autonomous motivation (PE)	-.050	-.114	-.069	-.256*^bd^
Past behaviour → Autonomous motivation (LT)	.136*^b^	.037	.147*^b^	.179
Past behaviour → PAS (Peer)	.067^abc^	.314**	.294**	.253*
Past behaviour → PAS (family)	-.038^abc^	.375**	.265**	.274*
Past behaviour → Attitude	.173*	.719**^bcd^	.165*	.235*
Past behaviour → Subjective norm	-.014	.564**^bcd^	.198*^d^	.237*^d^
Past behaviour → PBC	-.005	.182*^d^	.112	.188*^d^
Past behaviour → Intention	.501**^bc^	.471	.320**	.378**
Past behaviour → Behaviour	.087	.208*	.280**^c^	.078

*Note.* *R*^2^ Intention: Hungarian sample 49.6%; UK sample 51.1%; Romanian sample 42.7%; Slovakian sample 44.3%; overall (4 countries) 50.9%. *R*^2^ Behaviour: Hungarian sample 4.4%; UK sample 13.9%; Romanian sample 6.2%; Slovakian sample 7.2%; overall (4 countries) 7.8%. PAS = Perceived autonomy support; PE = Physical education context; LT = Leisure—time context; PBC = Perceived behavioural control.

^a^Significantly different from UK sample. ^b^Significantly different from Romanian sample. ^c^Significantly different from Slovakian sample. ^d^Significantly different from Hungarian sample.

*p < .05. **p < .001.

#### Relationships in the Model

Standardised path coefficients for the free parameters in the path analyses from the overall and each sample are provided in Table 2 The model accounted for 50.9%, 49.6%, 51.1%, 42.7% and 44.3% of the variance of leisure time physical activity intentions and 7.8%, 4.4%, 13.9%, 6.2% and 7.2% of the variance in physical activity behaviour in the overall, Hungarian, UK, Romanian and Slovakian samples, respectively.

**Figure 1:**
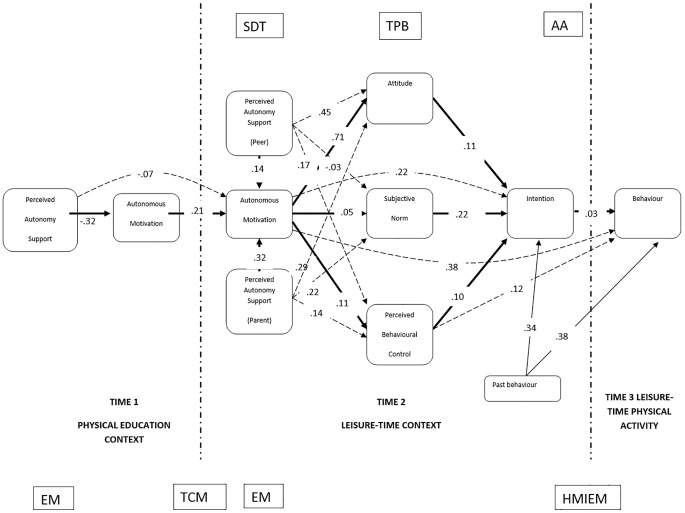
Antecedents and consequences: The extended trans-contextual model. *Note*. Solid lines illustrate the effects from the proposed motivational sequence that were tested in the model; broken lines indicate paths set to be free in order test indirect and mediation effects. SDT = Self-Determination Theory (SDT consists of three basic needs to satisfy, the need for *autonomy*, i.e., the belief that one is the origin and regulator of his or her own action, *competence*, i.e., the belief that one can efficaciously interact with the environment, and *relatedness*, i.e., the seeking and development of secure and connected relationships with others in one’s social context). TPB = Theory of Planned Behaviour (TPB shows the *attitude* that reflects an individual’s personal orientation toward engaging in the behaviour; *subjective norm* that reflects on social pressure from significant others regarding to the behaviour; *perceived behavioural control* that reflects on the impact of perceived abilities and barriers with respect to engaging in the behaviour. AA = Aetological Approach (AA shows *determinants of intentions*, decision making and physical activity *behaviour*). EM = Ecological Model (EM describes the interpersonal *determinants*, such as *social support*, from *family*, *friends* and *school*). TCM = Trans-Contextual Model (TCM explains the processes of how *autonomous motivation transfers across contexts*, and incorporates SDT, TPB and HMIEM). HMIEM = Hierarchical Model of Intrinsic and Extrinsic Motivation (HMIEM contends that motivation operates at *three levels*, first the *global* or personality, second the *contextual* or life domain and third, *situational* or state levels, also incorporates the fundamental tenets of SDT).

The model (Figure 1) contains the following observed, endogenous variables: perceived autonomy support from friends, perceived autonomy support from family/parents, attitude to exercise in leisure time, intention to exercise, current physical activity behaviour, perceived behaviour control (PBC), autonomous motivation in physical education, and autonomous motivation in leisure-time; and contains observed, exogenous variables: past physical activity behaviour and perceived autonomy support from teachers.

### Overall and Each Sample Analyses

For the overall sample (all four countries, Figure 1), the hypothesised model exhibited a good model fit with the data (CMIN = 67.84, *df* = 21, *p* = .001, NFI = .98, CFI = .99, RMSEA = .048, and GFI = .99). For Hungary, the hypothesised model also exhibited an acceptable model fit with the data (CMIN = 58.62, *df* = 21, *p* = .001, NFI = .97, CFI = .98, RMSEA = .068 and GFI = .97). For the UK the model also showed an acceptable model fit (CMIN = 47.011, *df* = 23, *p* = .002, NFI = .95, CFI = .97, RMSEA = .082 and GFI = .95). Regarding Slovakia, the model also demonstrated a good fit with the data (CMIN = 25.77, *df* = 18, *p* = .001, NFI = .98, CFI = .99, RMSEA = .042 and GFI = .98). For Romania, the model exhibited a good fit (CMIN = 30.54, *df* = 22, *p* = .01, NFI = .96 CFI = .99, RMSEA = .046 and GFI = .97).

## Discussion

Our study aimed to investigate the mechanism by which perceived autonomy support from three social agents (school/PE teachers, family and friends) influences autonomous motivation in physical education and leisure-time physical activity, what the link is between the two factors, and how these effects can generate physical activity intention leading to current physical activity behaviour. Ultimately, the study aim was to uncover why some people are physically active and others not, and whether there are any national or gender differences.

The AA explained the correlational relationships within the components of perceived autonomy support , as antecedents of autonomous motivation, and how these factors influenced intention and ultimately behaviour. The EM revealed the power of the social environment, and again, social support influenced young people’s intention to perform physical activity and current beehaviour. The TPB explained the volitional antecedents of intentional behaviour within attitude, subjective norm and PBC. SDT predicted the important role of autonomy, relatedness and competence in behavioural regulation. Vallerend’s HMM clarifyied the qualities of motivation on three levels: global—personality, contextual—life domain and state—situational level. Finally, the TCM revealed how autonomous motivation transfers across contexts (e.g., from school/educational to free living) andtherefore explained how leisure time behaviour is reinforced.

Physical education classes are supposed to instil behaviours and attitudes that promote physical activity in free-time and promote healthy lifestyle (Gonzales-Cutre, Cope, Harju, & Wuensch, 2014a). According to the overall sample in our study, autonomous motivation in PE was negatively correlated with most other variables. The rigorously structured curriculum in Central-Eastern European countries, as well as in some parts of the UK provides students with few options. Therefore, the expected function of physical education in schools fails to meet the criteria that lead to developing self-determined or autonomous motivation to young people’s leisure-time intention, and especially physical activity. Hein, Emeljanovas, and Mieziene (2016) found that Estonian and Lithuanian students perceive PE teachers’ behaviours as controlling, and even intimidating in some cases. However, many other studies (e.g., Hagger & Chatzisarantis, 2016) have found physical education teachers promote students’ autonomous motivation toward class activities by structuring the learning environment in favour of students. Self-regulated participation and engagement in activities can be achieved by fostering autonomous motivation in young people, who will take ownership over their actions, when they feel a sense of competence and satisfaction (Guay et al., 2010).

Our results, consistent with Hagger et al. (2009), confirm that perceived autonomy support from friends as well as family predicted autonomous motivation towards leisure-time physical activity across the four countries. However, Hungarian students received the most autonomy support from family compared with students from the other three countries, and more support from friends than those from Slovakia. Nevertheless, the effect of autonomy support from physical education teachers either had no or a negative effect on autonomous motivation in physical education classes, with the exception of UK students, who perceived more autonomy support from physical education teachers than the other three countries. These results support Gonzales-Cutre et al.’s (2014b) finding that autonomous motivation is transferred from physical education to leisure-time physical activity, and predicts attitudes, PBC and subjective norms, as well as forming intentions to participate in future physical activity. However, our findings support Hein et al. (2016), who reported controlling behaviour by PE teachers’ in some Eastern European countries. According to Martins (2015) a positive motivational climate should be created in physical education and sport to promote active lifestyles. Therefore, as Pakarinen et al. (2017) reported, positive motivational climate in physical activity can enhance physical and mental health benefits for young people.

Like Bagozzi and Kimmel (1995) and Chatzisarantis, Hagger, Smith, and Phoenix (2004), we investigated the relationship between past physical activity behaviour and intention as well as current physical activity. Our data revealed the strongest positive relationship between Hungarian and UK students’ past behaviour and intention followed by a moderate relationship in Slovakian and Romanian students’ past behaviour and intention. The relationship between past behaviour and current behaviour was moderate in UK and Romanian students, but weak in Hungarian and Slovakian students.

While previous work has examined national differences, this has usually been in the context of countries with similar cultures (eg Weinberg et al., 2000), unlike the individualistic and collectivist countries investigated in this study. Overall, students perceived more family support in collectivist countries than in individualistic countries. However, no other conclusive differences were found between cultures which implies that this factor may only have, at best, a weak impact on physical activity. However, all countries are (currently) part of the European Union and so may share values and behaviours.Comparisons between these countries and non-Western cultures may yield more widespread differences.

Regarding gender differences, girls reported more autonomy support both from family and friends than boys, furthermore girls reported higher autonomous motivation both in physical education and leisure time, while boys reported a higher level of current and past physical activity. These findings correspond with those observed by Gustafson and Rhodes (2006) and Telford, Telford, Olive, Cochrane, and Davey (2016). Furthermore, boys perceived more support from PE teachers compared to girls which is likely to influence their autonomous motivation in PE that can be transferred to free living, as another major determinant of current behaviour.Boys alsoperceived more autonomy support in physical education than girls. There was no significant difference between the intention of future physical activity behaviour in girls and boys. These results demonstrate the complexity of the casual relationships in behaviour in young people by explaining our findings within the TCM.

### Practical Recommendations

Our findings have several practical implications. We found that decision-making by students in relation to physical activity was determined by physical education teachers’ autonomy support and/or controlling motivating style (Amoura, Berjot, Gillet, Caruana, & Finez, 2015; Haerens et al., 2017). These interpersonal and environmental determinants were modified by social and autonomy support from family (parents, sisters/brothers) and friends. Understanding students’ autonomous motivation transfer through the TCM (Hagger et al., 2003) from a school context to leisure time can help educational and health practitioners to understand what influences young people’s behaviour. This has implications beyond physical activity, and can help understanding of why young people make a range of choices related to health.

Furthermore, clarifying the relationship between the influence of social agents (school, family friends) and students’ physical activity in collectivist as opposed to individualist countries can aid the development of educational materials and methods to orient young people to choose a healthy, physically active lifestyle across different nations.

### Limitations

Our study was correlational, and as such we cannot infer causal relationships between variables. We also focused our research on European countries which may have similar values and so differences between countries may be smaller than if we had obtained a more globally distributed sample.

### Future Work

An exploration of the relationship between autonomous motivation and perceived autonomy support in different countries is recommended, to clarify the relationship between these and different approaches to school physical education, in particular investigating the relationship between physical education teaching style and outcomes. Further work is also required to understand why the relationship between past behaviour and current behaviour is stronger in some countries than others. An examination of gender differences in outcomes would be beneficial for future work, as girls reported being less active than boys.

Exploration of the influence of teaching styles on physical activity also have potential for future study as these could provide practitioners with a greater understanding of the role they play in promoting physical activity in children outside the school environment. Further work is also needed to replicate the results of this study in relation to physical actvity behaviour in collectivist and individualistic countries, and also to examine whether these findings are replicable in a wider international sample.

### Conclusions

We found, like McDavid, Cox, and Amorose (2012), that parents and friends play significant roles in children’s motivation to participate in leisure-time physical activity. Furthermore, physical education teachers, although present at school only, still had an important role in supporting young people’s physical activity outside school. Furthermore, most physical education teachers (except those who employed a controlling style) still had an important role in supporting young people’s attitudes and behaviour related to physical activity outside school.

Overall, our study supports previous work regarding the relationship between young people’s perceived autonomy support in physical education and leisure- time and their leisure-time physical activity intention and behaviour by using the SDT, the TPB as well as the TCM. Findings also revealed a number of differences as well as similarities between collectivist countries (Hungary, Slovakia and Romania) and a country more orientated towards individualistic cultural norms (United Kingdom) (Markus & Kitayama, 1991). Our results also suggest that an optimal relationship should be developed between physical education teachers and students, and that autonomy support be maintained by family and friends to encourage students to pursue physical activity behaviour.

## References

[r1] Ajzen, I. (1985). *From intention to action: A Theory of Planned Behaviour*. In J. Kuhl & J. Beckman (Eds.), *Action control: From cognition to behaviour* (pp. 11–39). Heidelberg, Germany: Springer.

[r2] Ajzen I. (2002). Perceived behavioural control, self-efficacy, locus of control, and the Theory of Planned Behaviour. Journal of Applied Social Psychology, 32, 665–683. doi: 10.1111/j.1559-1816.2002.tb00236.x

[r3] Ajzen I. Madden T. J. (1986). Prediction of goal-directed behaviour: Attitudes, intentions and perceived behavioural control. Journal of Experimental Social Psychology, 22, 453–474. doi: 10.1016/0022-1031(86)90045-4

[r4] Ames C. Archer J. (1988). Achievement goals in the classroom: Students’ learning strategies and motivation processes. Journal of Educational Psychology 80, 260–267. doi: 10.1037/0022-0663.80.3.260

[r5] Amoura C. Berjot S. Gillet N. Caruana C. Finez L. (2015). Autonomy supportive and controlling styles of teaching. Swiss Journal of Psychology, 74 (3), 141–158. doi: 10.1024/1421-0185/a000156

[r35] Arbuckle, J. L. (2016). Amos (Version 24.0) [Computer software]. Chicago, IL, USA: IBM SPSS.

[r6] Armitage C. J. Conner M. (2001). Efficacy of the Theory of Planned Behaviour: A meta-analytic review. British Journal of Social Psychology, 40, 471–499. doi: 10.1348/014466601164939 11795063

[r7] Bagozzi R. P. Kimmel S. K. (1995). A comparison of leading theories for the prediction of goal directed behaviours. British Journal of Social Psychology, 34, 437–461. doi: 10.1111/j.2044-8309.1995.tb01076.x

[r8] Barkoukis V. Hagger M. S. Lampbropoulos G. Tsorbatzoudis H. (2010). Extending the Trans-Contextual Model in physical education and leisure-time contexts: Examining the role of basic psychological need satisfaction. British Journal of Educational Psychology, 80, 647–670. doi: 10.1348/000709910X487023 20175944

[r9] Baumann A. E. Seis R. S. Sallis J. F. Wells J. C. Loos R. J. F. Martin B. W. (2012). Correlates of physical activity: Why are some people physically active and others not? Lancet, 380, 258–271. doi: 10.1016/S0140-6736(12)60735-1 22818938

[r10] Bracken B. Barona A. (1991). State of the art procedures for translating, validating, and using psychoeducational tests in cross-cultural assessments. School Psychology International, 12, 119–132. doi: 10.1177/0143034391121010

[r11] Brislin, R. W. (1986). The wording and translation of research instruments. In W. J. Lonner & J. W. Berry (Eds.), *Field methods in educational research* (pp. 137–164). Newbury Park, CA, USA: Sage.

[r12] Chatzisarantis N. L. D. Hagger M. S. Smith B. (2007). Influences of perceived autonomy support on physical activity within the Theory of Planned Behaviour. European Journal of Social Psychology, 37, 934–954. doi: 10.1002/ejsp.407

[r13] Chatzisarantis N. L. D. Hagger M. S. Smith B. Phoenix C. (2004). The influences of continuation intentions on execution of social behaviour within the Theory of Planned Behaviour. British Journal of Social Psychology, 43, 551–583. doi: 10.1348/0144666042565399 15601509

[r14] Courneya K. S. McAuley E. (1994). Factors affecting the intention-physical activity relationship: Intention versus expectation and scale correspondence. Research Quarterly for Exercise and Sport, 65, 280–285. doi: 10.1080/02701367.1994.10607629 7973077

[r15] Deci, E. L., & Ryan, R. M. (1985). *Intrinsic motivation and self-determination in Human behaviour*. New York, NY, USA: Plenum Press.

[r16] Deci, E. L., & Ryan, R. M. (2002). *Handbook a self-determination research*. Rochester, NY, USA: University of Rochester Press.

[r17] Fattah A. Darwish E. Huber G. L. (2003). Individualism vs. collectivism in different cultures: A crosscultural study. Intercultural Education, 14(1), 47–55. doi: 10.1080/1467598032000044647

[r18] Godin G. Shephard R. J. (1985). A simple method to assess exercise behaviour in the community. Canadian Journal of Applied Sport Science, 10, 141–146. 4053261

[r19] Gonzales-Cutre D. Ferriz R. Bertran-Carrillo V. J. Andres-Fabra J. A. Montero-Carretero C. Cervello E. Moreno-Murcia J. A. (2014a). Promotion of autonomy for participation in physical activity: A study based on the Trans-Contextual Model of motivation. Educational Psychology: An International Journal of Experimental Educational Psychology, 34(3), 367–384. doi: 10.1080/01443410.2013.817325

[r20] Gonzales-Cutre D. Sicilia A. Beas-Jimenez M. Hagger M. S. (2014b). Broadening the Trans-Contextual Model of Motivation: A study with Spanish adolescents. Scandinavian Journal of Medicine and Sciences in Sports, 24, e306–e319. doi: 10.1111/sms.12142 24256054

[r21] Guay F. Chanal J. Ratelle C. F. Marsh H. W. Larose S. Boivin B. (2010). Intrinsic, identified, and controlled types of motivation for school subjects in young elementary school children. British Journal of Educational Psychology, 80, 711–735. doi: 10.1348/000709910X499084 20447334

[r22] Guay F. Mageau G. A. Vallerand R. J. (2003). On the hierarchical structure of self-determined motivation: A test of top-down and bottom-up effects. Personality and Social Psychology Bulletin, 29, 992–1004. 15189618 10.1177/0146167203253297

[r23] Gustafson S. L. Rhodes R. E. (2006). Parental correlates of physical activity in children and adolescents. Sports Medicine, 36(1), 79–97. doi: 10.2165/00007256-200636010-00006 16445312

[r24] Haerens L. Vanteenkiste M. De Meester A. Delrue J. Tallir I. Vande Broek G. (2017). Different combinations of perceived autonomy support and control: Identifying the most optimal motivating style. Physical Education and Sport Pedagogy, 23(1), 16–36. doi: 10.1080/17408989.2017.1346070

[r25] Hagger, M. S., & Chatzisarantis, N. L. D. (2007). The Trans-Contextual Model of motivation. In M. S. Hagger & N. L. D. Chatzisarantis (Eds.), *Intrinsic motivation and self-determination in exercise and sport* (pp. 53–70). Champaign, IL, USA: Human Kinetics.

[r26] Hagger M. S. Chatzisarantis N. L. D. (2016). The trans-contextual model of autonomous motivation in education: Conceptual and empirical issues and meta-analysis. Review of Educational Research, 86(2), 360–407. doi: 10.3102/0034654315585005 27274585 PMC4873731

[r27] Hagger M. S. Chatzisarantis N. L. D. Barkoukis V. Wang C. K. J. Baranowski J. (2005). Perceived autonomy support in physical education and leisure-time physical activity: A cross-cultural evaluation of the Trans-Contextual Model. Journal of Educational Psychology, 97(3), 376–390. doi: 10.1037/0022-0663.97.3.376

[r28] Hagger M. S. Chatzisarantis N. L. D. Culverhouse T. Biddle S. J. H. (2003). The process by which perceived autonomy support in physical education promotes leisure-time physical activity intentions and behaviour: A Trans-Contextual Model. Journal of Educational Psychology, 95(4), 784–795. doi: 10.1037/0022-0663.95.4.784

[r29] Hagger M. S. Chatzisarantis N. L. D. Hein V. Pihu M. Soós I. Karsai I. (2007). The perceived autonomy support scale for exercise settings (PASSES): Development, validity, and cross-cultural invariance in young people, Psychology of Sport and Exercise, 8(5), 632–653. doi: 10.1016/j.psychsport.2006.09.001

[r30] Hagger M. S. Chatzisarantis N. L. D. Hein V. Soós I. Karsai I. Lintunen T. Leemans S. (2009). Teacher, peer and parent autonomy support in physical education and leisure-time physical activity: A trans-contextual model of motivation in four nations. Psychology and Health, 24(6), 689–711. doi: 10.1080/08870440801956192 20205021

[r31] Hamilton K. M. White K. (2008). Extending the Theory of Planned Behavior: The role of self and social influences in predicting adolescent regular moderate-to-vigorous physical activity. Journal of Sport and Exercise Psychology, 30(1), 56–74. doi: 10.1123/jsep.30.1.56 18369243

[r32] Harris D. J. Atkinson G. (2009). International Journal of Sports Medicine – Ethical standards in sport and exercise science research. International Journal of Sports Medicine, 30(10), 701–702. doi: 10.1055/s-0029-1237378 19809942

[r33] Hein V. Emeljanovas A. Mieziene B. (2016). A cross-cultural validation of the controlling teacher behaviour scale in physical education. European Physical Education Review, 4, 1–16.

[r34] Hu L. Bentler P. M. (1999). Cut-off criteria for fit indexes in covariance structure analysis: Conventional criteria versus new alternatives. Structural Equation Modelling, 6, 1–55. doi: 10.1080/10705519909540118

[r36] Ingram K. L. Cope J. G. Harju B. L. Wuensch K. L. (2000). Applying to graduate school: A test of the Theory of Planned Behaviour. Journal of Social Behaviour and Personality, 15, 215–226.

[r37] Markus H. R. Kitayama S. (1991). Culture and the self: Implications for cognition, emotion and motivation. Psychological Review, 98, 224–253. doi: 10.1037/0033-295X.98.2.224

[r38] Martins, J. (2015). *Motivational climate in physical education and sport: How to promote active lifestyles, democracy and human rights?* Pestalozzi Training Resources, Council of Europe, pp. 1–23. Retrieved from https://www.coe.int/t/dg4/education/pestalozzi/Source/Documentation/TU/TU_SPORT_Martins.pdf

[r39] McDavid L. Cox A. E. Amorose A. J. (2012). The relative roles of physical education teachers and parents in adolescents’ leisure-time physical activity motivation and behaviour. Psychology of Sport and Exercise, 13, 99–107. doi: 10.1016/j.psychsport.2011.10.003

[r40] Mullan E. Markland D. Ingledew D. K. (1997). A graded conceptualisation of self-determination in the regulation of exercise behaviour: Development of a measure using confirmatory factor analysis. Personality and Individual Differences, 23, 745–752. doi: 10.1016/S0191-8869(97)00107-4

[r41] Pakarinen A. Parisod H. Smed J. (2017). Health game interventions to enhance physical activity self-efficacy of children: A quantitative systematic review. Journal of Advanced Nursing, 73(4), 794–811. doi: 10.1111/jan.13160 27688056

[r42] Pate R. R. Small M. L. Ross J. G. Young J. C. Flint K. H. Warren C. W. (1995). School physical education. Journal of School Health, 65, 312–318. doi: 10.1111/j.1746-1561.1995.tb03380.x 8558859

[r43] Ryan R. M. Connell J. P. (1989). Perceived locus of causality and internalization: Examining reasons for acting in two domains. Journal of Personality and Social Psychology, 57, 749–761. 2810024 10.1037//0022-3514.57.5.749

[r44] Ryan R. M. Deci E. L. (2000). Self-determination theory and the facilitation of intrinsic motivation, social development and well-being. American Psychologist, 55, 68–78. doi: 10.1037/0022-3514.57.5.749 11392867

[r45] Sallis J. F. Mckenzie T. L. (1991). Physical education’s role in public health. Research Quarterly for Exercise and Sport, 62, 124–137. doi: 10.1080/02701367.1991.10608701 1925034

[r46] Shephard R. Trudeau F. (2000). The legacy of physical education: Influences on adult lifestyle. Pediatric Exercise Science, 12, 34–50. doi: 10.1123/pes.12.1.34

[r47] Sherar L. B. Esliger D. W. Baxter-Jones A. D. G. Tremblay M. S. (2007). Age and gender differences in youth physical activity: Does physical maturity matter? Medicine and Science in Sport and Exercise, 6, 830–835. doi: 10.1249/mss.0b013e3180335c3c 17468582

[r49] Taylor W. Blair S. N. Cummings S. S. Wun C. C. Malina R. M. (1999). Childhood and adolescent physical activity patterns and adult physical activity. Medicine and Science in Sport and Exercise, 31, 118–123. doi: 10.1097/00005768-199901000-00019 9927019

[r48] Telford R. M. Telford R. D. Olive L. S. Cochrane T. Davey R. (2016). Why are girls less physically active than boys? Findings from the LOOK Longitudinal Study. PLoS One, 11(3), e0150041. 10.1371/journal.pone.0150041 26960199 PMC4784873

[r50] Vallerand R. J. (1997). Toward a hierarchical model of intrinsic and extrinsic motivation. Advances in Experimental Social Psychology, 29, 271–360. doi: 10.1016/S0065-2601(08)60019-2

[r51] Vallerand, R. J. (2001). *A hierarchical model of intrinsic and extrinsic motivation in sport and exercise*. In G. C. Roberts (Ed.), *Advances in motivation is sport and exercise* (pp. 263–320). Champaign, IL, USA: Human Kinetics.

[r52] Vallerand, R. J. (2007). A hierarchical model of intrinsic and extrinsic motivation for sport and physical activity. In M. S. Hagger & N. L. D. Chatzisarantis (Eds.), *Intrinsic motivation and self-determination in exercise and sport* (pp. 255–279). Champaign, IL, USA: Human Kinetics.

[r53] Vaz A. Pratley P. Alkire S. (2016). Measuring women’s autonomy in Chad using the Relative Autonomy Index. Feminist Economics, 22(1), 264–294. doi: 10.1080/13545701.2015.1108991

[r54] Weinberg R. Tenenbaum G. McKenzie A. Jackson S. Anshel M. Grove R. Fogarty G. (2000). Motivation for youth participation in sport and physical activity: Relationships to culture, self-reported activity levels, and gender. International Journal of Sport Psychology, 31, 321–346.

